# The metabolic score for insulin resistance in the prediction of major adverse cardiovascular events in patients after coronary artery bypass surgery: a multicenter retrospective cohort study

**DOI:** 10.1186/s13098-023-01133-7

**Published:** 2023-07-17

**Authors:** Shuai Zhang, Zhenguo Wu, Yifan Zhuang, Xiangfei Sun, Juan Wang, Sha Chen, Dachuan Guo, Panpan Xu, Cheng Zhang, Jianmin Yang

**Affiliations:** 1 National Key Laboratory for Innovation and Transformation of Luobing Theory, Shandong University, Jinan, 250012 China; 2 The Key Laboratory of Cardiovascular Remodeling and Function Research, Chinese Ministry of Education, Chinese National Health Commission and Chinese Academy of Medical Sciences, Shandong University, Jinan, 250012 China; 3 Department of Cardiology, Qilu Hospital of Shandong University, Jinan, 250012 China; 4 Department of Cardiovascular Surgery, Shandong Provincial Hospital, Cheeloo College of Medicine, Shandong University, Jinan, 250021 Shandong China; 5 Department of Cardiovascular Surgery, Shandong Provincial Hospital, Affiliated to Shandong First Medical University, Jinan, 250021 Shandong China; 6 Department of Cardiology, The Second Hospital of Shandong University, Jinan, 250033 Shandong China

**Keywords:** The metabolic score for insulin resistance, Coronary artery bypass graft surgery, Insulin resistance, Major adverse cardiovascular events, Prognosis

## Abstract

**Background:**

The metabolic score for insulin resistance (METS-IR) is a simple, convenient, and reliable marker for resistance insulin (IR), which has been regarded as a predictor of cardiovascular disease (CVD) and cardiovascular events. However, few studies examined the relationship between METS-IR and prognosis after coronary artery bypass graft (CABG). This study aimed to investigate the potential value of METS-IR as a prognostic indicator for the major adverse cardiovascular events (MACE) in patients after CABG.

**Method:**

1100 CABG patients were enrolled in the study, including 760 men (69.1%) and 340 women (30.9%). The METS-IR was calculated as Ln [(2 × FPG (mg/dL) + fasting TG (mg/dL)] × BMI (kg/m^2^)/Ln [HDL-C (mg/dL)]. The primary endpoint of this study was the occurrence of major adverse cardiovascular events (MACE), including a composite of all-cause death, non-fatal myocardial infarction (MI), coronary artery revascularization, and stroke.

**Result:**

The following-up time of this study was 49–101 months (median, 70 months; interquartile range, 62–78 months). During the follow-up period, there were 243 MACEs (22.1%). The probability of cumulative incidence of MACE increased incrementally across the quartiles of METS-IR (log-rank test, *p* < 0.001). Multivariate Cox regression analysis demonstrated a hazard ratio (95% CI) of 1.97 (1.36–2.86) for MACE in quartile 4 compared with participants in quartile 1. The addition of the METS-IR to the model with fully adjusting variables significantly improved its predictive value [C-statistic increased from 0.702 to 0.720, p < 0.001, continuous net reclassification improvement (NRI) = 0.305, < 0.001, integrated discrimination improvement (IDI) = 0.021, p < 0.001].

**Conclusion:**

METS-IR is an independent and favorable risk factor for predicting the occurrence of MACE and can be used as a simple and reliable indicator that can be used for risk stratification and early intervention in patients after CABG.

**Supplementary Information:**

The online version contains supplementary material available at 10.1186/s13098-023-01133-7.

## Introduction

Coronary artery disease (CAD) leads to the development of angina pectoris, myocardial infarction, sudden cardiac death, and ischemic heart failure, thus making cardiovascular disease (CVD) the leading cause of morbidity and mortality worldwide [1–3]. Coronary revascularization, including percutaneous coronary intervention (PCI) or coronary artery bypass graft (CABG) surgery, is an essential therapeutic option when managing patients with CAD and may also further reduce angina, improve quality of life, and increase survival [1, 4, 5]. CABG remains the gold-standard treatment for multivessel and left main coronary artery disease and then significantly improves cardiovascular outcomes. However, long-term survival after CABG remains poor [6, 7]

Therefore, it is imperative to identify and control the underlying risk factors for patients treated with CABG. Insulin resistance (IR) plays a critical role in many chronic diseases, including type 2 diabetes mellitus (T2DM) and CVD [8, 9]. Among adults, the global prevalence of IR ranges from 15.5 to 46.5% [10]. Meanwhile, metabolic risk factors hinder the control of morbidity and mortality in CAD, including post-CABG [11]. The gold standard for assessing IR is hyperinsulinemic-euglycemic clamp (HEC), an invasive, complex testing procedure and costly method; therefore, this technique is not commonly used in extensive epidemiological surveys [12].

The metabolic score for insulin resistance (METS-IR) is a reliable alternative diagnostic of IR and has a high concordance with the HEC [13]. So far, METS-IR has been closely associated with multiple CVD risk factors, such as diabetes, obesity, hypertension, arterial stiffness, hyperuricemia, and coronary artery calcification [13–16]. Our previous study found that METS-IR was associated with an increased risk of CVD in a 10-year cohort study [17]. A Korean cohort study also showed that elevated METS-IR predicted the risk of ischemic heart disease (IHD) in a community without diabetes and could be a valuable predictive marker for IHD [18].

Although several recent studies have demonstrated an association between METS-IR and CVD, no study has explored the relationship between METS-IR and postoperative prognosis after CABG. This study aimed to investigate the potential value of METS-IR as a prognostic indicator of major adverse cardiovascular events (MACE) in CAD patients after CABG and to provide primary care physicians with early screening for high-risk MACE and further close monitoring and intervention or possible potential value.

## Method

### Study population

**Fig. 1:**
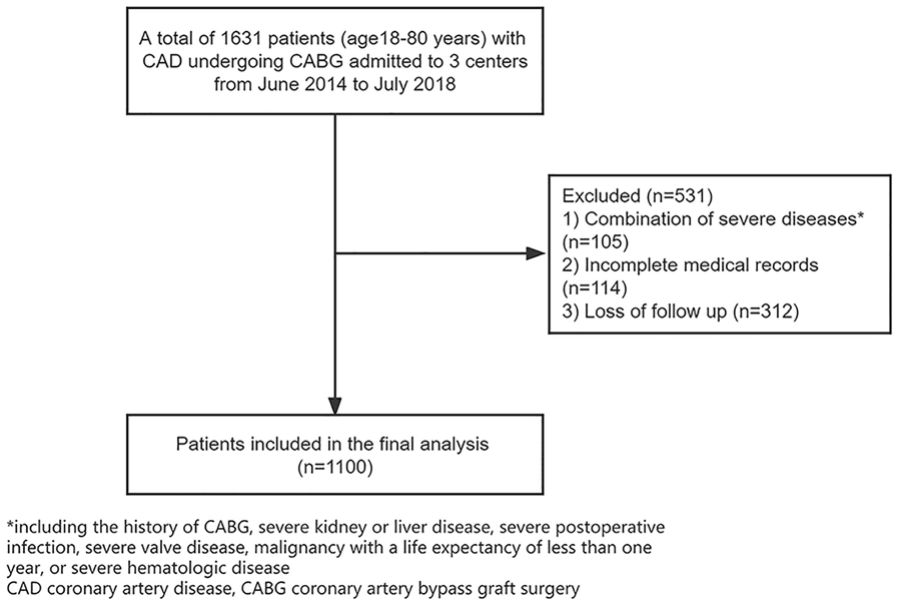
Flow diagram of patient selection

This study was a multicenter, retrospective cohort study. We retrospectively analyzed patients who underwent CABG from June 2014 to July 2018 at Qilu Hospital of Shandong University, Shandong Provincial Hospital, and The Second Hospital of Shandong University. Patients were excluded from the study if they had a combination of severe diseases; baseline data was incomplete and lost to follow-up. Eventually, 1100 patients involving 760 males (69.1%) and 340 females (30.9%) were enrolled in the study (Fig. 1).

This study complied with the Declaration of Helsinki and was approved by the Ethics Review Committee of Qilu Hospital of Shandong University, Shandong Provincial Hospital, and The Second Hospital of Shandong University. Since this was a retrospective cohort study and follow-up was conducted by telephone, the ethics committee allowed verbal consent.

### Data collection and definitions

Clinical data were collected from electronic medical records by trained clinicians who maintained confidentiality for the study. The patients' general status [age, sex, body mass index (BMI), left ventricular ejection fraction (LVEF), family history of CAD (FH-CAD) and myocardial infarction (MI) admission], medical history (previous MI, previous stroke, previous PCI, hypertension, DM and hyperlipidemia) was collected when patients are admitted. Morning fasting blood specimens were collected from patients within 24 h of admission and tested for [fasting plasma glucose (FPG), total cholesterol (TC), low-density lipoprotein cholesterol (LDL-C), high-density lipoprotein cholesterol (HDL-C), triglycerides (TG), serum creatinine (SCr) and uric acid (UA)]. The medication information [antiplatelet agents, statins, beta-blockers, angiotensin-converting enzyme inhibitors (ACEI)/angiotensin receptor blockers (ARB), and hypoglycemic agents (oral hypoglycemic agents or insulin)] when patients were discharged was also collected. BMI was defined as the weight (kg) divided by the square of height (m^2^). FH-CAD was defined as a first-degree relative with CAD aged less than 55 years for men and less than 65 years for women. Hypertension was defined when systolic blood pressure was ≥ 140 mmHg and/or diastolic blood pressure was ≥ 90 mmHg or when antihypertensive medication was used. DM was defined as fasting plasma glucose (FPG) level ≥ 26 mg/dL (7.0 mmol/L) or 2-h plasma glucose level ≥ 200 mg/dL (11.1 mmol/L) after oral glucose tolerance test (OGTT) or use of oral hypoglycemic agents or insulin [19]. Hyperlipidemia was defined as ICD-10 code E78 with lipid-lowering medication or total serum cholesterol ≥ 240 mg/dL) [20]. The estimated glomerular filtration rate (eGFR) is calculated from SCr. The eGFR (mL/min/1.73m^2^) = 175 × SCr (mg/dL) ^− 1.234^ × age (year) ^− 0.179^ × 0.79 (in the case of women) [21]. METS-IR was calculated as Ln [(2 × FPG (mg/dL) + fasting TG (mg/dL)] × BMI (kg/m^2^)/Ln [HDL-c (mg/dL)] [13].

### Endpoint definition

The primary endpoint of this study was the occurrence of MACE, including a composite of all-cause death (cardiovascular or non-cardiovascular death), non-fatal MI, coronary artery revascularization (CABG or PCI), and stroke. For patients with multiple clinical events, MACE was defined as recording the first event and time of occurrence and included in the analysis. The secondary endpoints were all-cause death, non-fatal MI, coronary artery revascularization, and stroke. The diagnosis of non-fatal MI was according to the Fourth Universal Definition of Myocardial Infarction [22].

### Statistical analysis

Statistical analysis was performed using SPSS version 25.0 (SPSS, Chicago, IL) and R software version 4.2.0 (R Foundation for Statistical Computing). Continuous variables were presented as mean ± standard deviation (SD) for normally distributed continuous variables, which compared the difference between groups using the Student’s t-test or ANOVA test or median with the 25th and 75th percentiles for non-normally distributed continuous variables, which reached the difference between groups using Mann–Whitney U test or Kruskal–Wallis H test. Categorical variables were expressed with counts and percentages and compared using the chi-square test or Fisher exact test. We used Pearson or Spearman correlation analysis to evaluate the association between the METS-IR and cardiovascular risk factors. Kaplan–Meier analysis was conducted in R language to plot the MACE incidence curves, and the *p*-value was tested by the log-rank test. The cumulative number of MACE occurrences over the months of follow-up was also plotted. Variables were analyzed by univariate Cox regression analysis. We then built three prediction models using multivariate Cox regression analysis to see whether METS-IR was an independent predictor of MACE occurrence: model 1 was adjusted for age and gender, and model 2 was a partially adjusted model that included variables with *p* < 0.10, including age, hypertension, DM, previous stroke, LVEF, eGFR, TC, LDL-C, and beta-blockers. Model 3 was a fully adjusted model including age, gender, previous MI, previous stroke, previous PCI, MI admission, hypertension, DM, hyperlipidemia, FH-CAD, TC, LDL-C, eGFR, LVEF, antiplatelet agents, beta-blockers, statins, ACEI/ARB, and hypoglycemic agents. Also, METS-IR were input into the model analysis as a continuous and categorical variable (quartiles of METS-IR). Bias in the results due to multicollinearity was avoided by calculating the variance inflation factor (VIF) of the variables in the model. We found no evidence of covariance in the model, as all VIFs were < 10. METS-IR was further converted into a standardized variable in the model to identify the predictive value per SD increase. We also conducted a subgroup analysis based on age, gender, hypertension, hyperlipidemia, DM, and FH-CAD to determine whether the association between METS-IR and MACE differed across subgroups and to calculate the *p*-value for the interaction. In addition, we performed multivariate Cox regression analyses using fully adjusted models for the incidence of all-cause mortality, non-fatal MI, coronary revascularization, and stroke. We calculated the C-index, continuous net reclassification (NRI), and integrated discrimination improvement (IDI) in models 2 and 3, with and without METS-IR. Finally, in a series of sensitivity analyses, we excluded patients with a history of lipid-lowering or glucose-lowering drug use and patients with non-cardiovascular death. All statistical tests were two-sided; A *p*-value less than 0.05 was considered statistically significant.

## Result

### Baseline characteristics

The baseline data between the included and excluded groups were shown in Additional file 1: Table S1, and no statistical differences were found between the two groups except for age, LVEF, previous MI, and LDL-C. A total of 1100 individuals with a mean age of 62.84 ± 8.28 years with complete follow-up information were included in our study, of whom 760 (69.1%) were men. The baseline characteristics of patients with or without MACE were shown in Table 1. Summarily, patients with events were more likely to be older (*p* = 0.001) and had less LVEF (*p* = 0.002). Statistical significance was also found in FBG (*p* < 0.001), TC (*p* < 0.001), LDL-C (*p* = 0.008), HDL-C (*p* = 0.012), TG (*p* < 0.001), eGFR (*p* = 0.022), UA (*p* = 0.009), and beta-blockers (*p* < 0.001). In addition, subjects with MACE had a higher level of METS-IR than those without event (41.73 ± 7.53 vs. 39.52 ± 6.63, *p* < 0.001). No difference between groups was found in the traditional causative factors of coronary heart disease, such as hypertension, DM, and hyperlipidemia (Table 1). Then, patients were divided into four groups based on the quartiles of METS-IR (Quartile 1 < 35.3, n = 276; 35.3 ≤ Quartile 2 < 39.6, n = 277; 39.6 ≤ Quartile3 < 44.5, n = 276; Quartile4 ≥ 44.5, n = 271). We found that the variables, including METS-IR, age, BMI, DM, FPG, HDL-C, TG, UA, and hypoglycemic drugs, were statistically different. For the primary and second endpoints, MACE, non-fatal MI, and stroke showed statistical significance between groups (Table 2).

**Table 1:** Baseline characteristics of the study population according to the occurrence of MACE

Variables	Total (n = 1100)	Without event (n = 857)	With event (n = 243)	*p*-value
*General conditions*				
Age (years)	62.84 ± 8.28	62.41 ± 8.29	64.33± 8.10	**0.001**
Male, n (%)	760 (69.1)	596 (69.5)	164 (67.5)	0.582
BMI (kg/m^2^)	25.66 ± 3.63	25.55 ± 3.55	26.02 ± 3.88	0.075
LVEF (%)	60.00 (54.00–65.00)	60.00 (55.00–65.00)	60.00 (50.00–65.00)	**0.002**
FH-CAD, n (%)	215 (19.6)	166 (19.4)	49 (20.2)	0.783
Admission for MI, n (%)	197 (17.9)	155 (18.1)	42 (17.3)	0.778
*Medical history, n (%)*				
Previous MI	215 (19.5)	160 (18.7)	55 (22.6)	0.170
Pervious stroke	165 (15.0)	121 (14.1)	44 (18.1)	0.128
Previous PCI	114 (10.4)	91 (10.6)	23 (9.5)	0.636
Hypertension	693 (63.0)	529 (61.7)	164 (67.5)	0.114
DM	363 (33.0)	273 (31.9)	90 (37.0)	0.142
Hyperlipidemia	372 (33.8)	283 (33.0)	89 (36.6)	0.318
*Laboratory text*				
FBG (mmol/L)	5.36 (4.75–6.85)	5.25 (4.70–6.59)	5.73 (4.85–8.30)	** < 0.001**
TC (mmol/L)	4.27 ± 1.13	4.18 ± 1.24	4.58 ± 1.04	** < 0.001**
LDL-C (mmol/L)	2.45 (1.93–3.04)	2.42 (1.90–2.96)	2.59 (2.01–3.31)	**0.008**
HDL-C (mmol/L)	1.13 ± 0.26	1.14 ± 0.25	1.09 ± 0.28	**0.012**
TG (mmol/L)	1.32 (0.98–1.76)	1.25 (0.97–1.69)	1.49 (1.13–2.05)	** < 0.001**
eGFR (ml/min/1.73m^2^)	106.74 ± 30.04	107.84 ± 30.54	102.85 ± 27.91	**0.022**
UA (µmol/L)	303.00 (256.00–361.00)	299.00 (254.00–356.00)	314.00 (266.75–378.25)	**0.009**
*Cardiovascular medications, n (%)*				
Antiplatelet drugs	1089 (99.0)	849 (99.1)	240 (98.8)	0.715
Statins	898 (81.6)	695 (81.1)	203 (83.5)	0.400
Beta-blockers	973 (88.5)	775 (90.4)	198 (81.5)	** < 0.001**
ACEI/ARB	172 (15.6)	129 (15.1)	43 (17.7)	0.318
Hypoglycemic drugs	262 (23.8)	203 (23.7)	59 (24.3)	0.865
METS-IR	40.01 ± 6.90	39.52 ± 6.63	41.73 ± 7.53	** < 0.001**

Continuous variables were presented as mean ± SD or median (interquartile range) and categorical variables were expressed with number (proportion, %)

*MACE* major adverse cardiovascular events, *BMI* body mass index, *LVEF* left ventricle ejection fraction, *FH-CAD* family history of coronary artery disease, *MI* myocardial infarction, *PCI* percutaneous transluminal coronary intervention, *DM* diabetes mellitus, *FPG* fasting plasma glucose, *TC* total cholesterol, *LDL-C* low-density lipoprotein-cholesterol, *HDL-C* high-density lipoprotein-cholesterol, *TG* triglyceride, *eGFR* estimated glomerular filtration rate, *UA* uric acid, *ACEI* angiotensin-converting enzyme inhibitors, *ARB* angiotensin receptor blockers, *METS-IR* the metabolic score for insulin resistance

*p* values in bold are < 0.05

**Table 2:** Baseline characteristics in patients with CABG surgery based on quartiles of METS-IR

Variables	Quartile 1 (N = 276)	Quartile 2(N = 277)	Quartile 3 (N = 276)	Quartile 4 (N = 271)	*p*-value
METS-IR	32.30 (29.93–33.74)	37.54 (36.48–38.60)	41.80 (40.71–43.28)	48.15 (45.98–51.22)	** < 0.001**
*General conditions*					
Age (years)	64.65 ± 7.80	62.82 ± 8.05	61.95 ± 7.93	61.90 ± 9.05	** < 0.001**
Male, n (%)	171 (62.0)	190 (68.6)	196 (71.0)	203 (74.9)	0.010
BMI (kg/m^2^)	21.66 ± 2.04	24.57 ± 1.48	26.54 ± 1.93	29.93 ± 2.59	** < 0.001**
LVEF (%)	60.00 (53.00–66.00)	60.00 (55.00–65.00)	60.00 (53.00–65.00)	60.00 (53.00–65.00)	0.195
FH-CAD, n (%)	47 (17.1)	66 (23.8)	53 (19.2)	49 (18.1)	0.202
Admission for MI, n (%)	46 (16.7)	46 (16.6)	57 (20.7)	48 (17.7)	0.566
*Medical history, n (%)*					
Previous MI	51 (18.5)	54 (19.5)	61 (22.1)	49 (18.1)	0.633
Pervious stroke	41 (14.9)	39 (14.1)	41 (14.9)	44 (16.2)	0.915
Previous PCI	23 (8.3)	26 (9.4)	32 (11.6)	33 (12.2)	0.404
Hypertension	160 (58.0)	168 (60.6)	185 (67.0)	180 (66.4)	0.074
DM	63 (22.8)	74 (26.7)	103 (37.3)	123 (45.4)	** < 0.001**
Hyperlipidemia	83 (30.1)	88 (31.8)	103 (37.3)	98 (36.2)	0.219
*Laboratory text*					
FBG (mmol/L)	5.04 (4.48–5.74)	5.20 (4.63–6.38)	5.54 (4.83–6.99)	6.09 (4.96–8.45)	** < 0.001**
TC (mmol/L)	4.24 (3.58–5.11)	4.05 (3.45–4.85)	4.00 (3.49–5.02)	4.07 (3.44–4.90)	0.164
LDL-C (mmol/L)	2.48 (1.87–3.02)	2.36 (1.93–2.95)	2.52 (2.03–3.16)	2.44 (1.90–3.02)	0.382
HDL-C (mmol/L)	1.25 (1.09–1.43)	1.13 (1.01–1.27)	1.07 (0.94–1.19)	1.00 (0.87–1.14)	** < 0.001**
TG (mmol/L)	1.06 (0.81–1.38)	1.22 (0.95–1.68)	1.43 (1.09–1.68)	1.50 (1.18–2.07)	** < 0.001**
eGFR(ml/min/1.73m^2^)	107.64 ± 26.03	106.50 ± 26.16	106.59 ± 26.18	106.20 ± 39.80	0.949
UA (µmol/L)	295.29 ± 77.03	309.8 ± 85.08	309.25 ± 83.64	335.95 ± 96.24	** < 0.001**
*Cardiovascular medications, n (%)*					
Antiplatelet drugs	275 (99.6)	271 (97.8)	275 (98.7)	268 (98.9)	0.090
Statins	219 (79.3)	235 (84.8)	224 (81.2)	220 (81.2)	0.401
Beta-blockers	250 (90.6)	247 (89.2)	240 (87.0)	236 (87.1)	0.482
ACEI/ARB	33 (12.0)	41 (14.8)	43 (15.6)	55 (20.3)	0.059
Hypoglycemic drugs	43 (15.6)	51 (18.4)	68 (24.6)	100 (36.9)	** < 0.001**
*Outcomes, n (%)*					
MACE	48 (17.4)	51 (18.4)	62 (22.5)	82 (30.3)	**0.001**
All-cause death	19 (6.9)	15 (5.4)	18 (6.5)	22 (8.1)	0.654
Coronary artery revascularization	11 (4.0)	13 (4.7)	16 (5.8)	18 (6.6)	0.513
Non-fatal MI	14 (5.1)	14 (5.1)	15 (5.4)	27 (10.0)	**0.047**
stroke	8 (2.9)	14 (5.1)	19 (6.9)	24 (8.9)	**0.022**
Cardiovascular death	6 (2.2)	7 (2.5)	7 (2.5)	10 (3.7)	0.718

Continuous variables were presented as mean ± SD or median (interquartile range) and categorical variables were expressed with number (proportion, %)

*METS-IR* the metabolic score for insulin resistance, *MACE* major adverse cardiovascular events, *BMI* body mass index, *LVEF* left ventricle ejection fraction, *FH-CAD* family history of coronary artery disease, *MI* myocardial infarction, *PCI* percutaneous coronary intervention, *DM* diabetes mellitus, *FPG* fasting plasma glucose, *TC* total cholesterol, *LDL-C* low-density lipoprotein-cholesterol, *HDL-C* high-density lipoprotein-cholesterol, *TG* triglyceride, *eGFR* estimated glomerular filtration rate, *UA* uric acid, *ACEI* angiotensin-converting enzyme inhibitors, *ARB* angiotensin receptor blockers, *MACE* major adverse cardiovascular events

*p* values in bold are < 0.05

### Correlations between the METS-IR score and cardiovascular risk factors

Pearson or Spearman correlation analysis was used to assess the correlation between METS-IR and traditional cardiovascular risk factors, and the results were shown in Table 3. METS-IR was positively correlated with BMI, UA, TG, and FPG (*p* < 0.05) and negatively associated with age, HDL-C, and LVEF (*p* < 0.05) (Table 3).

**Table 3:** Correlations between the METS-IR and cardiovascular risk factors

Variables	Correlation coefficient (r)	*p*-value
Age (years)	− 0.105^&^	**0.001**
BMI (kg/m^2^)	0.885^&^	** < 0.001**
HDL-C (mmol/L)	− 0.425^&^	** < 0.001**
UA (µmol/L)	0.170^&^	** < 0.001**
eGFR (ml/min/1.73m^2^)	− 0.027^&^	0.377
TC (mmol/L)	− 0.041^&^	0.170
LVEF (%)	− 0.062*	**0.040**
TG (mmol/L)	0.354*	** < 0.001**
FPG (mmol/L)	0.269*	** < 0.001**

*METS-IR* the metabolic score for insulin resistance, *BMI* body mass index, *HDL-C* high-density lipoprotein-cholesterol, *UA* uric acid, *eGFR* estimated glomerular filtration rate, *TC* total cholesterol, *LVEF* left ventricle ejection fraction, *TG* triglyceride, *FPG* fasting plasma glucose

*p* values in bold are < 0.05

^&^Person

*Spearman

### Univariate Cox regression analyses for MACE

The following-up time of this study was 49–101 months (median, 70 months; interquartile range, 62–78 months). During our follow-up period, there were 243 MACEs (22.1%). Meanwhile, 74 (6.7%) all-cause death, 70 (6.4%) non-fatal MI, 58 (5.3%) coronary artery revascularization, and 65 (5.9%) stroke were recorded. The association between MACE and variables was shown in Table 4. We observed that age, DM, LVEF, TC, eGFR, beta-blockers, and METS-IR had statistically significant correlations with the incidence of MACE (*p* < 0.05). The unadjusted HR (95% CI) for the incidence of MACE with per SD increase in the METS-IR was 1.36 (1.20–1.54) (Table 4).

**Table 4:** Univariate Cox regression analyses for MACE

Variables	HR	HR95%CI	*p*-value
Male	0.86	0.66–1.13	0.288
Age	1.03	1.02–1.05	** < 0.001**
Previous MI	1.17	0.87–1.58	0.308
Previous stroke	1.32	0.96–1.84	0.092
Previous PCI	0.87	0.56–1.33	0.516
Admission for MI	0.82	0.59–1.16	0.262
Hypertension	1.27	0.97–1.66	0.086
DM	1.30	1.01–1.69	**0.049**
Hyperlipidemia	1.16	0.90–1.51	0.256
FH-CAD	0.95	0.70–1.31	0.770
LVEF	0.16	0.05–0.47	**0.001**
TC	1.22	1.12–1.33	** < 0.001**
eGFR	0.99	0.99–1.00	**0.006**
LDL-C	1.14	1.00–1.31	0.057
Antiplatelet drugs	0.88	0.28–2.74	0.820
Beta-blockers	0.46	0.33–0.63	** < 0.001**
Statins	1.20	0.85–1.68	0.303
ACEI/ARB	1.06	0.76–1.48	0.736
Hypoglycemic drugs	1.15	0.86–1.53	0.348
METS-IR	1.05	1.03–1.06	** < 0.001**
MEST-IR (Per SD)	1.36	1.20–1.54	** < 0.001**

*MACE* the major adverse cardiovascular events, *HR* hazard ratio, *CI* confidence interval, *MI* myocardial infarction, *PCI* percutaneous transluminal coronary intervention, *DM* diabetes mellitus, *FH-CAD* family history of coronary artery disease, *LVEF* left ventricle ejection fraction, *TC* total cholesterol, *eGFR* estimated glomerular filtration rate, *LDL-C* low-density lipoprotein-cholesterol, *ACEI* angiotensin-converting enzyme inhibitors, *ARB* angiotensin receptor blockers, *METS-IR* the metabolic score for insulin resistance

*p* values in bold are < 0.05

### The risk of primary and secondary endpoints by METS-IR quartiles

**Fig. 2:**
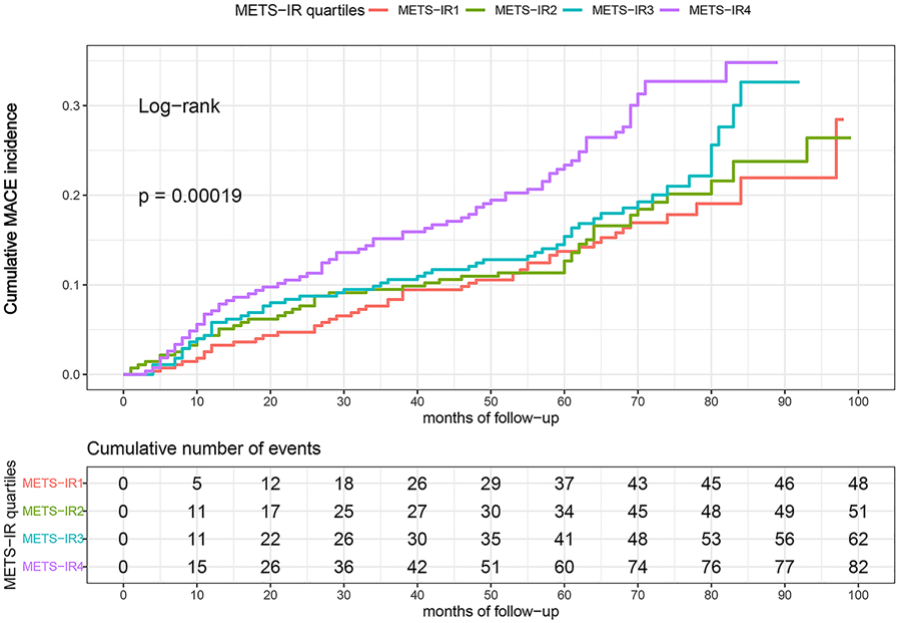
Kaplan–Meier survival curves for MACE across the METS-IR quartiles. The cumulative incidence of MACE during follow-up grouped according tothe METS-IR quartile was analyzed by Kaplan–Meier curves. The *p*-value was calculated with the log-rank test. *MACE* major adverse cardiovascular events, *METS-IR* the metabolic score for insulin resistance

**Fig. 3:**
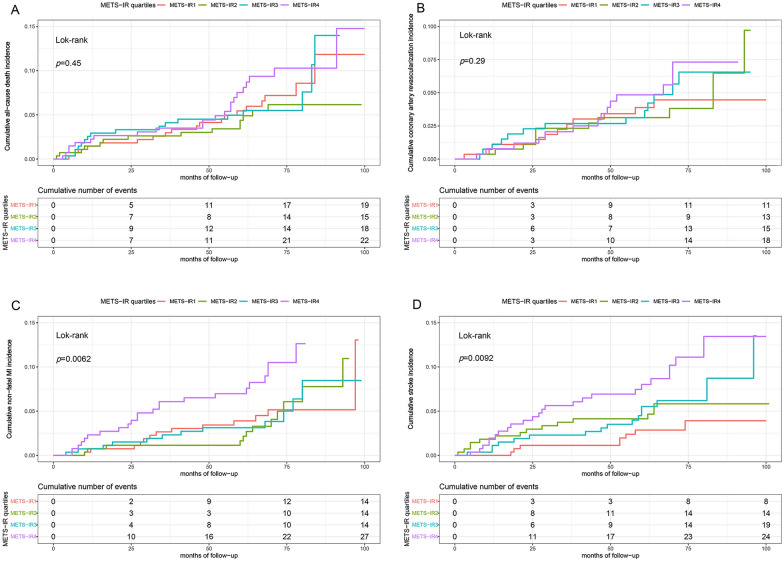
Kaplan–Meier survival curves for all-cause death (**A**), coronary artery revascularization (**B**), non-fatal MI (**C**), and stroke (**D**) across the METS-IR quartiles. The cumulative incidence of all-cause death (**A**), coronary artery revascularization (**B**), non-fatal MI (**C**), and stroke (**D**)) during follow-up according to the METS-IR quartile grouping was analyzed by Kaplan–Meier curves. The *p*-value was calculated with the log-rank test. *METS-IR* the metabolic score for insulin resistance, *MI* myocardial infraction

The probability of cumulative incidences of MACE increased incrementally across the quartiles of METS-IR (log-rank test, *p* < 0.001) (Fig. 2). Although no statistical significances were found between all-cause death, coronary artery revascularization and METS-IR quartiles (Fig. 2A, B). Statistical significances existed between non-fatal MI, stroke, and METS-IR quartiles (*p* = 0.0062, *p* = 0.0092, respectively) (Fig. 3C, D). Multivariate Cox regression analysis of the three models for MACE were shown in Table 5. For per unit increase in METS-IR, HR (95% CI) of incidence of MACE was 1.05 (1.03–1.07), 1.04 (1.03–1.06), and 1.05 (1.03–1.07) in model 1, 2, and 3 while per SD increase in METS-IR was respectively 1.40 (1.24–1.60), 1.35 (1.19–1.53) and 1.36 (1.20–1.55). The risk for MACE of quartile 3 and quartile 4 increased by 47% (HR = 1.47, 95% CI 1.00–2.14) and 119% (HR = 2.19, 95% CI 1.53–3.14) in model 1, compared to quartile 1. A similar pattern was observed in model 2 and model 3. The risk for MACE of quartile 4 increased by 94% (HR = 1.94, 95% CI 1.34–2.79) and 97% (HR = 1.97, 95% CI 1.36–2.86). The increased risk of MACE from quartile 1 to quartile 4 in Models 1, 2, and 3 was statistically significant (*p* for the trend in three models < 0.001) (Table 5).

**Table 5:** Multivariate Cox regression analyses for MACE

MTES-IR	Incident MACE, n (%)	HR (95% CI)		
		Model1	Model2	Model3
Per 1 Unit increase	243 (22.1%)	**1.05 (1.03–1.07)** **	**1.04 (1.03–1.06)** **	**1.05 (1.03–1.07)** **
Per 1 SD increase		**1.40 (1.24–1.60)** **	**1.35 (1.19–1.53)** **	**1.36 (1.20–1.55)** **
Quartile 1	48 (17.4%)	1 (Reference)	1 (Reference)	1 (Reference)
Quartile 2	51 (18.4%)	1.12 (0.75–1.66)	1.09 (0.73–1.62)	1.08 (0.72–1.60)
Quartile 3	62 (22.5%)	**1.47 (1.00–2.14)** *	1.34 (0.91–1.97)	1.34 (0.91–1.98)
Quartile 4	82 (30.3%)	**2.19 (1.53–3.14)** **	**1.94 (1.34–2.79)** **	**1.97 (1.36–2.86)** **
*p* for trend		** < 0.001**	** < 0.001**	** < 0.001**

Model 1: adjusted for age and gender

Model 2: adjusted for variables with *p*-value < 0.10, including age, hypertension, DM, previous stroke, LVEF, eGFR,   TC, LDL-C, beta-blockers

Model 3: adjusted for age, gender, previous MI, previous stroke, previous PCI, admission for MI, hypertension, DM, hyperlipidemia, FH-CAD, TC, LDL-C, eGFR, LVEF, antiplatelet drugs, beta-blockers, statins, ACEI/ARB and hypoglycemic drugs

*MACE* the major adverse cardiovascular events, *METS-IR* the metabolic score for insulin resistance, *HR* hazard ratio, *CI* confidence interval, *SD* standard deviation

*p* values in bold are < 0.05

^*^p < 0.05

^**^p < *0.001*

Then we analyzed the predictive value of METS-IR on secondary endpoints, including all-cause death, non-fatal MI, coronary artery revascularization, and stroke (Table 6). The risk for non-fatal MI and stroke in quartile 4 increased by 114% [2.14 (1.08–4.22)] and 256% [3.56 (1.56–8.15)], compared with quartile 1. In all-cause death and coronary artery revascularization, METS-IR was not an independent factor for MACE. In addition, to further verify the relationship between MTES-IR and MACE, we study two groups of individuals (group 1: Excluding patients with a history of lipid-lowering or hypoglycemic using; group 2: Excluding non-cardiovascular death) in Additional file 1: Table S2.

**Table 6:** Multivariate Cox regression analyses for composite of all cause death, non-fatal MI, coronary artery, revascularization and stroke

METS-IR	HR (95% CI)			
	Composite of all-cause death	Non-fatal MI	Coronary artery revascularization	Stroke
Per 1 unit increase	1.03 (1.00–1.07)	**1.05 (1.01–1.09)** *	**1.05 (1.01–1.09)** *	**1.06 (1.02–1.10)** *
Per 1 SD increase	1.23 (0.97–1.55)	**1.37 (1.07–1.77)** *	**1.36 (1.03–1.79)** *	**1.46 (1.14–1.80)** *
Quartile 1	1 (Reference)	1 (Reference)	1 (Reference)	1 (Reference)
Quartile 2	0.77 (0.38–1.55)	0.99 (0.47–2.11)	1.09 (0.48–2.47)	1.56 (0.64–3.77)
Quartile 3	1.12 (0.57–2.23)	0.95 (0.44–2.03)	1.26 (0.56–2.84)	2.23 (0.96–5.21)
Quartile 4	1.33 (0.69–2.55)	**2.14 (1.08–4.22)** *	1.79 (0.82–3.92)	**3.56 (1.56–8.15)** *
*p* for trend	0.276	**0.027**	0.122	**0.001**

Adjusted for age, gender, previous MI, previous stroke, previous PCI, admission for MI, hypertension, DM, hyperlipidemia, FH-CAD, TC, LDL-C, eGFR, LVEF, antiplatelet drugs, beta-blockers, statins, ACEI/ARB and hypoglycemic drugs

*METS-IR* the metabolic score for insulin resistance, *MI* myocardial infarction, *HR* hazard ratio, *CI* confidence interval, *SD* standard deviation

*p* values in bold are < 0.05

*p < 0.05

### Subgroup analysis

**Fig. 4:**
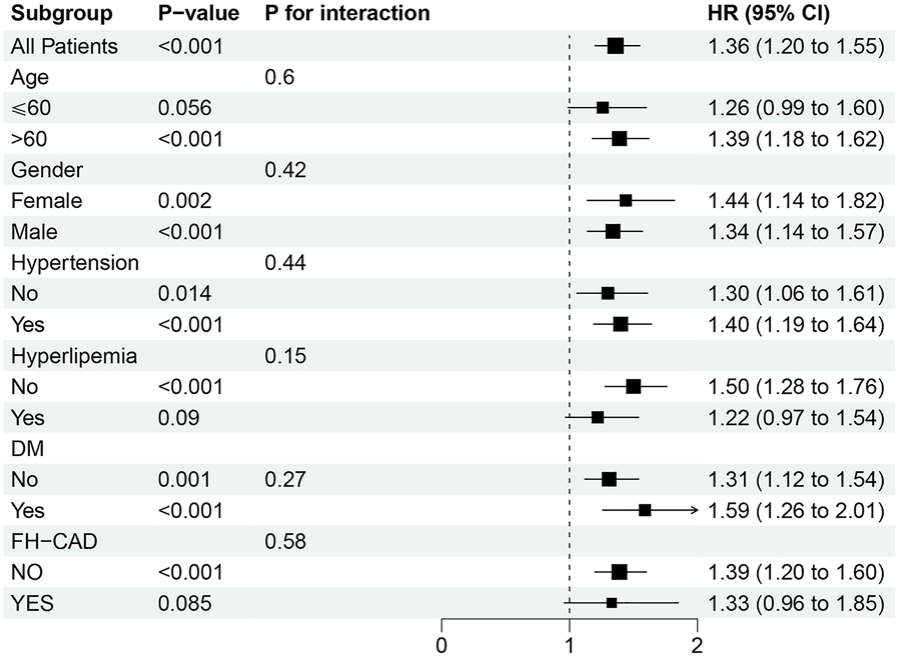
Subgroup and interaction between the METS-IR (Per SD) and MACE across various subgroups *MACE* major adverse cardiovascular events, *METS-IR* the metabolic score for insulin resistance, *DM* diabetes mellitus, *FH-CAD* family history of coronary artery disease

The association between METS-IR and MACE was examined in the subgroup analysis, and the *p*-value for interaction was calculated in Fig. 4. No significant interaction was found between subgroups and the METS-IR for incident MACE in the fully adjusted model (Model 3). Statistical significance was observed among patients aged > 60 years, without hyperlipidemia and without FH-CAD. In addition, we further analyzed the subgroups of non-fatal MI, stroke, and METS-IR (per SD) (Additional file 1: Table S3).

### Evaluation of the predictive performance of the METS-IR for MACE

As shown in Table 7, C-statistic, NRI, and IDI were calculated to evaluate the incremental predictive value of METS-IR for MACE in model 2 and model 3. Risk prediction was increased by adding METS-IR to model 2, with the C-statistic increase rising from 0.692 to 0.711 (*p* < 0.001) for MACE. NRI and IDI analysis showed statically significant improvement in prediction value [continuous NRI (95% CI): 0.266 (0.125–0.408), *p* < 0.001, IDI (95% CI): 0.020 (0.010–0.029), *p* < 0.001]. Also, adding METS-IR to model 3 could significantly improve outcome prediction [C-statistic increased from 0.702 to 0.720, *p* < 0.001, continuous NRI = 0.305, *p* < 0.001, IDI = 0.021, *p* < 0.001] (Table 7).

**Table 7:** The incremental predictive value of the METS-IR for MACE in the model 2 and model 3

	C-statistic (95%CI)	*p*-value	Continuous NRI (95%CI)	*p*-value	IDI (95%CI)	*p*-value
Model2 without METR-IR	0.692 (0.637–0.747)	** < 0.001**	Ref		Ref	
Model2 with METR-IR	0.711 (0.677–0.745)		0.266 (0.125–0.408)	** < 0.001**	0.020 (0.010–0.029)	** < 0.001**
Model3 without METR-IR	0.702 (0.667–0.737)	** < 0.001**	Ref		Ref	
Model3 with METR-IR	0.720 (0.686–0.754)		0.305 (0.164–0.446)	** < 0.001**	0.021 (0.011–0.031)	** < 0.001**

*METS-IR* the metabolic score for insulin resistance, *MACE* major adverse cardiovascular events, *NRI* net reclassification improvement, *IDI* integrated discrimination improvement, *Ref.* reference

*p* values in bold are < 0.05

## Discussion

To our knowledge, this was the first study examining the relationship between METS-IR and MACE after CABG. There were several key findings from our research as follow: (1) METS-IR was significantly associated with the occurrence of MACE, non-fatal MI, and stroke after CABG, independent of traditional cardiovascular risk factors; (2) The significant association between METS-IR and MACE was mainly observed among that age > 60 years, without hyperlipidemia and without FH-CAD; (3) the addition of the METS-IR to the traditional risk model significantly improved its predictive value. Taken together, our current study proved the predictive value of the METS-IR for MACE after CABG.

CABG is the most durable and complete treatment of IHD [23]. Despite significant improvement in cardiovascular outcomes, in the post-CABG period, the progression of atherosclerosis in the grafted vessels and the original diseased vessels can promote the recurrence of ischemic events [23]. Preventing recurrent cardiovascular events in patients who underwent CABG is a significant challenge [24]. The previous study reported that the incidence of 5-year MACE in individuals with post-CABG is around 11.8 to 31.0% [25], indicating that early identification of patients prone to MACE after having CABG is essential. However, most previous studies focused on the effect of traditional risk factors [6, 7]. Still, the metabolic burden of patients is high [26], and there is insufficient evidence for the prognostic impact of METS-IR on CABG.

IR is associated with incident CAD and MACE, independent of traditional cardiovascular risk factors [27–29]. The gold standard for assessing IR is HEC. However, HEC is costly, time-consuming, invasive, and requires trained epidemiological or large-scale intervention study staff. METS-IR, a non-insulin-based insulin resistance, was found to be a higher concordance with HEC and has been proven to be associated with multiple risk factors of CVD and cardiovascular events [13–17]. There has been no research on the correlation between METS-IR and the prognosis of patients who underwent CABG.

Previous studies have shown that METS-IR is related to the severity of coronary lesions in CAD patients and the incidence of CVD events [30, 31]. METS-IR had an excellent predictive value for IHD from a longitudinal study among Korean without diabetes [18]. A cohort study of 18,609 hypertensive individuals revealed a nearly J-shaped association between METS-IR and the risk of stroke and ischemic stroke [32]. A previous study showed that patients receiving CABG had a higher incidence of stroke than those receiving PCI [33]. Stroke and MI remain significant causes of morbidity following CABG. In the current study, we found that the higher METS-IR was strongly associated with individuals’ occurrence of MACE, non-fatal MI, and stroke after CABG. IR is a risk factor for MI and stroke development and is also associated with poor prognosis [34, 35]. Therefore, early detection and control of IR may contribute to the early prevention of MI and stroke after CABG.

In patients with CAD, cardiovascular medications, such as hypoglycemic, antiplatelet, and lipid-lowering drugs, may affect laboratory parameters level testing involving METS-IR calculation. However, some studies proved that METS-IR was still an independent factor of CAD after adjusting for the drugs' effect [17, 31]. Our current study is consistent with the previous survey. By adjusting for drug use, we found a 1.97-fold increase in the incidence of MACE for the highest METS-IR compared with the lowest METS-IR.

In the subgroup analysis, the significant correlation between METS-IR and MACE was found to occur in people aged > 60 years, without hyperlipidemia and without FH-CAD. We did not find a positive correlation between MACE and FH-CAD, possibly because the disease-promoting effect of FH-CAD masked the role of IR surrogate index indicators in our data. In addition, patients with hyperlipidemia taking lipid-lowering medications may affect METS-IR, weakening the prediction of MACE.

Previous studies found that adding METS-IR to traditional risk prediction models could predict the occurrence of CVD, coronary heart disease (CHD), and stroke [17, 36]. However, when combined with traditional risk factors, the enhancement of METS-IR in improving the prediction of MACE after CABG was unclear. In the present research, adding METS-IR to the fully adjusted model had a remarkable incremental predictive value for predicting MACE in post-CABG patients, with an increment of 0.018 in C-statistic (*p* < 0.001), NRI of 30.5% (*p* < 0.001), and IDI of 2.1% (*p* < 0.001). These findings suggest that METS-IR can be used clinically for risk stratification after CABG.

There are several limitations of this study that are worth considering. First, because this study was a post hoc cohort study analysis, our findings should be interpreted as hypothesis-generating rather than conclusive. Second, we collected the primary endpoint by telephone follow-up, which leaves the possibility of bias in the patient recall. Third, METS-IR is dynamic, and we did not collect and study changes in METS-IR on outcomes during follow-up. Finally, to ensure the accuracy of data analysis, we excluded patients with a combination of severe diseases, incomplete baseline data, and loss of follow-up, resulting in only 66% of the original dataset being included in the analysis, which might influence the results and produce biased estimates. In order to clarify the impact of high lost follow-up rates on the research results, we compared the baseline data of the excluded and included populations. We found no statistical difference between the two groups except for age, LVEF, previous MI, and LDL-C variables. This suggested that the excluded population populations might not remarkably affect the validity of the current results. Further prospective studies should be conducted and confirm our findings.

## Conclusion

In summary, METS-IR is significantly associated with the occurrence of MACE after CABG and is a valuable predictor of MACE. Therefore, we propose METS-IR as a simple and reliable indicator for clinical work on risk stratification and early intervention in patients who underwent CABG.

## Supplementary Information

**Additional file 1: Table S1.** Baseline characteristics between excluded and included participants. **Table S2.** Sensitivity analysis for the association between the METS-IR and MACE. **Table S3.** Subgroup and interaction between the METS-IR (Per SD) and non-fatal MI and across various subgroups.

## Data Availability

The datasets used and/or analyzed during the current study are available from the corresponding author on reasonable request.

## Abbreviations

METS-IR: The metabolic score for insulin resistance
CVD: Cardiovascular disease
MACE: Major adverse cardiovascular events
MI: Myocardial infraction
CAD: Coronary artery disease
PCI: Percutaneous coronary intervention
CABG: Coronary artery bypass graft surgery
T2DM: Type 2 diabetes mellitus
NRI: Continuous net reclassification improvement
IDI: Integrated discrimination improvement
BMI: Body mass index
LVEF: Left ventricular ejection fraction
FH-CAD: Family history of CAD
FPG: Fasting plasma glucose
TC: Total cholesterol
LDL-C: Low-density lipoprotein cholesterol
HDL-C: High-density lipoprotein cholesterol
TG: Triglycerides
SCr: Serum creatinine
UA: Uric acid
ACEI: Angiotensin-converting enzyme inhibitors
ARB: Angiotensin receptor blockers
OGTT: Oral glucose tolerance test
eGFR: Estimated glomerular filtration rate
SD: Standard deviation
VIF: Variance inflation factor
HR: Hazard ratio
CI: Confidence interval
HEC: Hyperinsulinemic-euglycemic clamp
