# Supplementary material for: The metabolic score for insulin resistance in the prediction of major adverse cardiovascular events in patients after coronary artery bypass surgery: a multicenter retrospective cohort study
Source: Diabetol Metab Syndr. 2023 Jul 17;15:157. doi: 10.1186/s13098-023-01133-7 (PMC10351175; doi:10.1186/s13098-023-01133-7)
Supplement: Supplementary file 1 — Additional file 1: Table S1. Baseline characteristics between excluded and included participants. Table S2. Sensitivity analysis for the association between the METS-IR and MACE. Table S3. Subgroup and interaction between the METS-IR (Per SD) and non-fatal MI and across various subgroups. [file 13098_2023_1133_MOESM1_ESM.docx]

Table S1. Baseline characteristics between excluded and included participants

| Characteristics | Excluded  (N=531) | Included  (N=1100) | *p* value |
| --- | --- | --- | --- |
| General conditions |  |  |  |
| Age (years) | 61.82±8.42 | 62.84±8.28 | 0.021 |
| Male, n (%) | 370 (69.7) | 760 (69.1) | 0.819 |
| BMI (kg/m^2^) | 25.65±2.89 | 25.66±3.63 | 0.905 |
| LVEF (%) | 60.00 (51.00-64.00) | 60.00 (54.00-65.00) | 0.001 |
| Admission for MI, n (%) | 102 (19.2) | 197 (17.9) | 0.539 |
| Medical history, n (%) |  |  |  |
| Previous MI | 46 (8.7) | 215 (19.5) | 0.038 |
| Pervious stroke | 82 (15.4) | 165 (15.0) | 0.825 |
| Previous PCI | 51 (9.6) | 114 (10.4) | 0.662 |
| Hypertension | 314 (59.1) | 693 (63.0) | 0.142 |
| DM | 160 (30.1) | 363 (33.0) | 0.258 |
| Hyperlipidemia | 184 (34.7) | 372 (33.8) | 0.780 |
| Laboratory text |  |  |  |
| FBG (mmol/L) | 5.39 (4.64-6.69) | 5.36 (4.75-6.85) | 0.269 |
| TC (mmol/L) | 4.23±1.10 | 4.26±1.13 | 0.498 |
| LDL-C (mmol/L) | 2.64 (2.17-3.39) | 2.45 (1.93-3.04) | ＜0.001 |
| HDL-C (mmol/L) | 1.11±0.52 | 1.14±0.25 | 0.374 |
| TG (mmol/L) | 1.28 (0.96-1.72) | 1.32 (0.98-1.76) | 0.335 |
| eGFR (ml/min/1.73m^2^) | 103.87±25.89 | 106.74±30.04 | 0.064 |
| UA (µmol/L) | 304.00 (257.00-364.00) | 303.00 (256.00-361.00) | 0.791 |
| Cardiovascular medications,  n (%) |  |  |  |
| Antiplatelet drugs | 520 (98.3) | 1089 (99.0) | 0.236 |
| Statins | 419 (79.4) | 898 (81.6) | 0.282 |
| Beta-blockers | 463 (87.5) | 973 (88.5) | 0.623 |
| ACEI/ARB | 100 (19.0) | 172 (15.6) | 0.089 |
| Hypoglycemic drugs | 109 (22.3) | 262 (23.8) | 0.521 |
| METS-IR | 39.87±6.58 | 40.01±6.90 | 0.236 |

Excluded participants including the combination of severe diseases, incomplete baseline data, and loss of follow-up

*p* values in bold are＜0.05

Table S2 Sensitivity analysis for the association between the METS-IR and MACE

| METS-IR | HR (95% CI) | |
| --- | --- | --- |
|  | Group 1 | Group 2 |
| Per 1 Unit increase | **1.04 (1.01-1.06) **** | **1.05 (1.03-1.07) ***** |
| Per 1 SD increase | **1.27 (1.08-1.51) **** | **1.28 (1.07-1.53) **** |
| Quartile 1 | 1 (Reference) | 1 (Reference) |
| Quartile 2 | 1.48 (0.88-2.48) | 1.15 (0.75-1.76) |
| Quartile 3 | **1.72 (1.03-2.89) *** | 1.40 (0.93-2.12) |
| Quartile 4 | **1.99 (1.18-3.34) *** | **2.13 (1.44-3.17) ***** |
| *p* for trend | **0.008** | **＜0.001** |

Group 1: Excluding patients with a history of lipid-lowering or hypoglycemic usage.

Group 2: Excluding non-cardiovascular death.

**p*＜0.05

***p*＜0.01

*** *p*＜0.001

*p* values in bold are＜0.05

Table S3 Subgroup and interaction between the METS-IR (Per SD) and non-fatal MI and across various subgroups

| Subgroup | Non-fatal MI |  | Stroke | |  |
| --- | --- | --- | --- | --- | --- |
|  | HR (95%CI) | *p* for interaction | HR (95%CI) | *p* for interaction | |
| Age |  | 0.778 |  | 0.977 | |
| ≤60 | 1.40 (0.91-2.16) |  | 1.48 (0.90-2.44) |  | |
| ＞60 | 1.33 (0.97-1.83) |  | **1.46 (1.06-2.00) *** |  | |
| Sex |  | 0.557 |  | 0.164 | |
| Female | 1.28 (0.76-2.13) |  | **1.89 (1.25-2.85) **** |  | |
| Male | **1.45 (1.08-1.95) *** |  | 1.26 (0.90-1.76) |  | |
| Hypertension |  | 0.254 |  | **0.031** | |
| NO | 1.41 (0.89-2.24) |  | **2.18 (1.38-3.42) **** |  | |
| YES | **1.56 (1.13-2.14) **** |  | 1.16 (0.82-1.62) |  | |
| DM |  | 0.064 |  | 0.250 | |
| NO | 1.12 (0.81-1.54) |  | 1.36 (0.99-1.87) |  | |
| YES | **2.38 (1.45-3.91) **** |  | **1.88 (1.14-3.10) *** |  | |
| Hyperlipidemia |  | 0.092 |  | 0.923 | |
| NO | **1.69 (1.24-2.31) **** |  | **1.45 (1.04-2.03) *** |  | |
| YES | 0.96 (0.58-1.59) |  | **1.62 (1.07-2.46) *** |  | |

**p*＜0.05

***p*＜0.01

*p* values in bold are＜0.05
